# Higher Neuromuscular Manifestations of Fatigue in Dynamic than Isometric Pull-Up Tasks in Rock Climbers

**DOI:** 10.1515/hukin-2015-0059

**Published:** 2015-10-14

**Authors:** Gennaro Boccia, Luisa Pizzigalli, Donato Formicola, Marco Ivaldi, Alberto Rainoldi

**Affiliations:** 1 Motor Science Research Center, School of Exercise & Sport Sciences, SUISM, Department of Medical Sciences, University of Turin, Italy.; 2 CeRiSM Research Center “Sport, Mountain, and Health”, Rovereto (TN), Italy.

**Keywords:** electromyography, brachioradialis, teres major, rock climbing, fatigue

## Abstract

Neuromuscular assessment of rock climbers has been mainly focused on forearm muscles in the literature. We aimed to extend the body of knowledge investigating on two other upper limb muscles during sport-specific activities in nine male rock climbers. We assessed neuromuscular manifestations of fatigue recording surface electromyographic signals from brachioradialis and teres major muscles, using multi-channel electrode arrays. Participants performed two tasks until volitional exhaustion: a sequence of dynamic pull-ups and an isometric contraction sustaining the body at half-way of a pull-up (with the elbows flexed at 90°). The tasks were performed in randomized order with 10 minutes of rest in between. The normalized rate of change of muscle fiber conduction velocity was calculated as the index of fatigue. The time-to-task failure was significantly shorter in the dynamic (31 ±10 s) than isometric contraction (59 ±19 s). The rate of decrease of muscle fiber conduction velocity was found steeper in the dynamic than isometric task both in brachioradialis (isometric: −0.2 ±0.1%/s; dynamic: −1.2 ±0.6%/s) and teres major muscles (isometric: −0.4±0.3%/s; dynamic: −1.8±0.7%/s). The main finding was that a sequence of dynamic pull-ups lead to higher fatigue than sustaining the body weight in an isometric condition at half-way of a pull-up. Furthermore, we confirmed the possibility to properly record physiological CV estimates from two muscles, which had never been studied before in rock climbing, in highly dynamic contractions.

## Introduction

In recent years research on rock climbing has increased. However, neuromuscular investigation conducted on rock climbers has been mainly focused on forearm muscles and handgrip/finger strength and fatigability (Esposito et al., 2009; MacLeod et al., 2007; Quaine and Vigouroux, 2004; Quaine et al., 2003). Surface electromyography (sEMG) has been used to extract useful information upon neuromuscular strategies adopted in climbing. Beyond “global signal” information such as the amplitude and the mean frequency of the power spectrum, multi- channel sEMG techniques now offer the possibility to estimate muscle fiber conduction velocity (CV) within the detection muscle volume. CV is an important physiological variable defined as the propagation’s speed of the motor unit action potential along the sarcolemma (Merletti et al., 1990) and it is related to the size of the recruited muscle fibers (Blijham et al., 2006; Farina et al., 2004). Regarding highly dynamic conditions, such as those occurring in rock climbing, the non-stationarity structure of sEMG signals should be taken into account by estimating CV using appropriate signal processing (Farina and Merletti, 2000; Farina et al., 2000; Farina et al., 2004).

The aforementioned sEMG variables have been used to characterize neuromuscular fatigue during isometric (Gonzalez-Izal et al., 2012) and dynamic tasks (Farina et al., 2004). We referred to myoelectric manifestations of fatigue as all the changes in sEMG variables occur during sustained muscle contractions. Myoelectric manifestations of fatigue are due to changes in muscle fiber membrane excitability and motor units action potentials propagation and are related to alteration of muscle metabolic conditions and failure of excitation-contraction coupling (Brody et al., 1991). The changes are believed to be related to a decrease in muscle pH (Brody and Pollock, 1991) and are reflected by decrements of CV and mean power spectral frequency (MNF), and increments of average rectified value (ARV) (Merletti and Knaflitz, 1990). In particular, greater recruitment of type II muscle fibers has been demonstrated to generate greater myoelectric manifestations of fatigue, measured as a greater decrease of MNF and CV over time (Merletti et al., 1999; Gonzalez-Izal et al., 2012).

Rock climbing involves intense alternate periods of isometric and dynamic muscle contractions. However, the fatigability of upper limbs muscles other than forearm muscles has not been investigated for this type of contractions. For this reason, the aim of the study was to highlight neuromuscular activation strategies and manifestations of fatigue during two types of contractions until volitional exhaustion: the first was an isometric contraction; the second was a series of pull-ups. A further aim was to verify the possibility to properly record and extract information from multichannel sEMG signals during these kinds of exercises by two muscles, which seem essential in climbing: brachioradialis and teres major.

## Material and Methods

### Participants

Nine male medium-high level rock climbers were recruited for the study. The age, body height, and body mass of the participants (mean ±SD) were respectively 31±5 years, 1.75±0.10 m, and 65 ±6 kg. Participants declared their technical ability to make routes as top-rope on crags according to the French scale. Self-reported grades were ranged from 7a to 8a. In the French rating scale, technical sub-grades are expressed as a number and letters (for example 6a, 6b, 6c), starting from grade 4 increasing until the maximum difficulty of 9b. All subjects provided written informed consent to participate. This study was approved by the ethics committee of the University of Turin and was performed in accordance with the Declaration of Helsinki.

### Procedure

**Figure 1:**
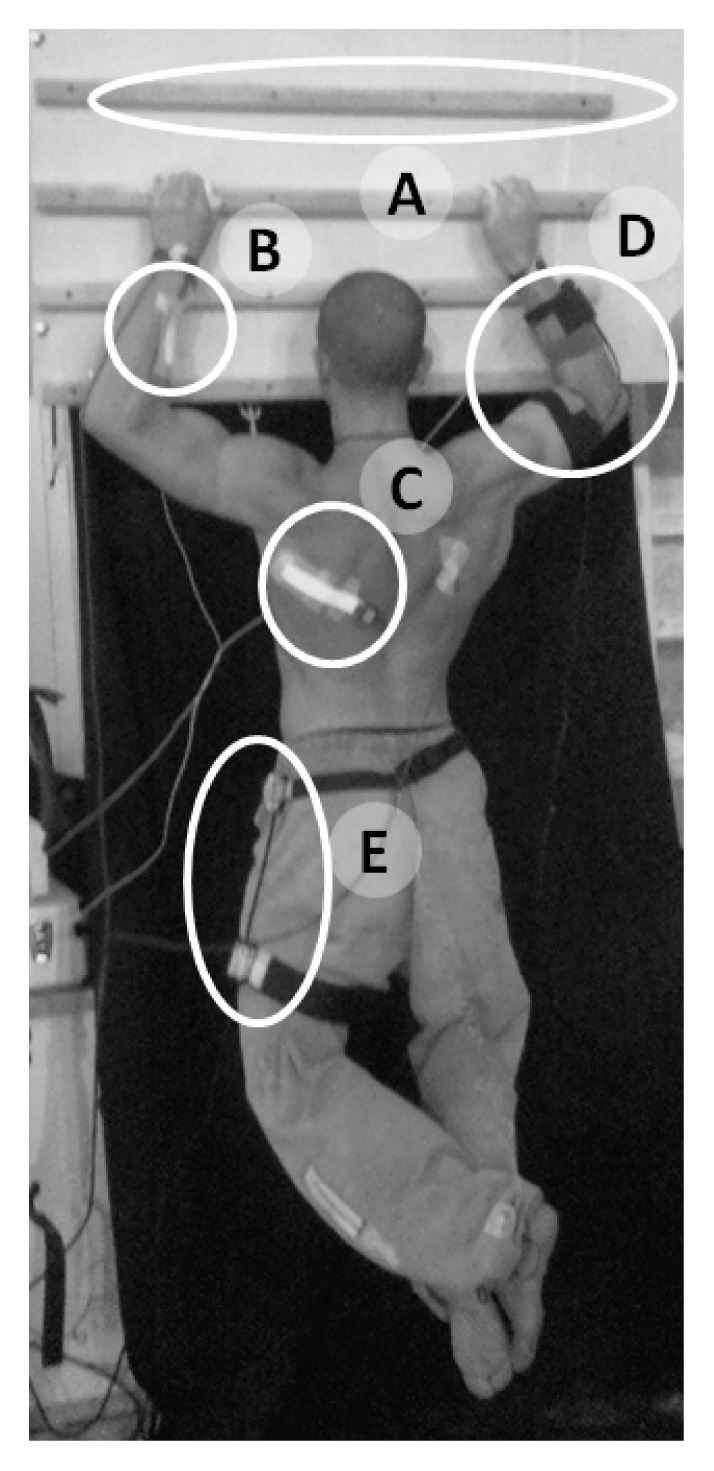
Experimental setup: climbers performed pull-ups hanged on a A) Pan Gullich bar; two linear arrays of electrodes were positioned on B) brachioradialis and C) teres major muscles; two electrogoniometers were fixed to the D) elbow and to the E) hip

Participants were asked to refrain from performing strenuous physical activity in the 24 hours before the experimental session. Before measurements, they performed a self-guided warm up that included few repetitions of pull-ups. After the warm up they rested for 5 minutes and then they performed the two tasks in randomized order, with 10 minutes of rest in between. One task was an isometric contraction (ISO) during which participants were requested to remain hanged at half-way of a pull up (with the elbows flexed at 90° and the arms abducted at 90°), sustaining the body weight until exhaustion (Figure 1). The second task was a sequence of pull-ups until volitional exhaustion (END) at a frequency freely chosen by the participant. The exercise started with the participant in a hanging position and the arms extended. In each cycle of pull-ups, participants were requested to move the chin above the hands and return with arms fully extended. One investigator visually inspected the correct executions of the exercises. The tasks were performed on a campus board: a training tool that allows a finger grip only and that is commonly used by climbers to strengthen fingers and arms (Figure 1A), in a sport specific way.

### Measures

Surface EMG was recorded from *brachioradialis* (Figure 1B) and *teres major* (Figure 1C) muscles using adhesive linear arrays (OT Bioelettronica, Torino, Italy) of eight electrodes (silver bars 5×1 mm with 5 mm of interelectrode distance). The two muscles were chosen as the first acting over the elbow and the second over the shoulder. In particular *brachioradialis* was chosen for its great importance in the elbow flexion when the grip is in neutral position (Bressel et al., 2001), as in the adopted setup. The skin was slightly abraded with abrasive paste and cleaned with water before electrodes placement. The optimal position and orientation of the array were sought and selected for each muscle, on the basis of the visual inspection of the sEMG signals as expressed in Piitulainen et al.’s study (Piitulainen et al., 2013). The main innervation zones of both muscles were identified prior to the 8-electrode-array placement. This was done using a dry linear array of 16 electrodes (silver bar electrodes with 5 mm inter-electrode distance, OT Bioelettronica, Torino, Italy). During this search for the optimal location for the 8-electrode-array, sEMG signals were recorded and displayed online on a computer screen while the participant produced submaximal isometric contractions of each muscle separately. This procedure was repeated from various parts of both muscles until the sites with clear muscle fiber action potential propagation and the main innervation zones were identified. The arrays were then placed parallel to muscle fibers, either proximally or distally with respect to the main innervation zone location, depending on anatomical features of the participant, where unidirectional propagation of the motor unit action potentials was detected. To avoid the innervation zone or tendon moving under the electrodes during the movement, the optimal position of the arrays was checked for both the maximum extension and flexion angles (Rainoldi et al., 2000). Moreover, all EMG channels were visually inspected offline not to overlap with the innervation zone for the entire range of motion, and if this occurred these channels were rejected from the analysis.

The sEMG signals were amplified, bandpass filtered (3-dB bandwidth, 10–500 Hz, 12 dB/oct slope on each side), sampled at 2048 samples/s per channel and converted to digital data by a 12-bit A/D converter (EMG64, 64 channel amplifier; OT Bioelettronica, Torino, Italy). They were displayed in real time, and stored on the disk of a personal computer. The signals were acquired in single differential configuration.

Two electrogoniometers (MLTS700, ADinstruments, New Zealand) were used to acquire the joint angles during the exercises: one goniometer was fixed to the elbow in the contralateral arm in relation to the electrodes (Figure 1D), to trigger contraction phases, and the other one was fixed to the hip (Figure 1E). The goniometer on the hip has been used to track compensatory hip movements.

### Data management

In the dynamic task, the angle signal from the goniometer on the elbow joint was used to identify the pull-up repetitions and the concentric (CON) and eccentric (ECC) phases of the movement. The range of motion (ROM) of the elbow joint was calculated, for each cycle, as the difference between the angle reached in maximum extension and the angle reached in maximum flexion. Cycle length was calculated as the time interval between two consecutive maximum extensions. The mean angular velocity of the elbow joint was calculated as ROM/cycle length. The amplitude of hip movements was calculated as the ARV of the hip angle signal. When tights and trunk were aligned (as in the starting position) the hip angle was considered as 0°.

The sEMG signals were visually inspected in order to select the best channels to use for sEMG variable estimations. The three single differential signals with higher similarity and propagation were used to estimate muscle fiber CV (Rainoldi et al., 1999). CVs were estimated using two adjacent double differential signals (based on one triplet of single differential signals). ARV and MNF were estimated on the central of three channels used for CV estimates (Rainoldi and Galardi, 1999).

With regard to the isometric contractions, the sEMG variables were estimated on non-overlapping epochs of 250 ms (Merletti et al., 2003). In dynamic contractions, to test the variability of muscle activation through the elbow angles, the sEMG variables were estimated, for both CON and ECC phases, on three time instants corresponding to 25, 50, and 75% of the maximum ROM of the elbow angle (where 100% corresponded to maximum elbow flexion). Hence sEMG variables were estimated six times for each cycle of pull-ups, three in CON and three in ECC (Figure 3). ARV and MNF were estimated for each cycle of contraction on epochs 125 ms long centred in the six instants identified. Epoch length of 125 ms was chosen on the base of the work of Farina and Merletti (2000) where 125 ms were determined to be the shortest reliable epoch length within non-stationary signals. CV was estimated with the method described in Farina et al. (2004) as the average of CV of the motor unit action potentials occurring close to the selected time instants. The method is based on the estimation of the delay between the two double differential signals weighted by a Gaussian window of which standard deviation was set to 40 ms. This method has been developed to specifically estimate CV during explosive contractions.

The comparison between ECC and CON phases with respect to ISO were performed using the 125 ms epoch centered at the 50% of ROM of dynamic contractions. Noisy channels and signals with CV estimates beyond the physiological range (2–6 m/s) (Merletti, Farina 2003) were excluded, if any.

### Statistical analysis

Linear regression analysis was applied to the time course of sEMG and kinematic variable estimates. The initial value of variables was calculated as the y-axis intercept (the beginning of the task) of regression lines. Then the normalized rate of change (normalized slope) was calculated as the percentage ratio between the slope of regression line and its initial value.

Shapiro-Wilk tests showed non-parametric distributions of data. Wilcoxon signed rank tests were used to compare time-to-task-failure between ISO and END. One sample Wilcoxon signed rank test was used to test whether the normalized slopes of elbow angular velocity were different from zero. Friedman’s tests (and successive Dunn’s post-hoc tests) were used to check for differences in sEMG variables among three conditions: ISO; END CON; END ECC. Friedman tests (and successive Dunn’s post-hoc tests) were also used to study the influence of the elbow angle (at 25, 50, and 75% of ROM) on the sEMG variables for each muscle, task, and contraction phase. The level of significance was set at p=0.05. Data are all expressed as mean ±SD.

## Results

All sEMG recorded signals showed almost three consecutive single differential signals (a so called “triplet”), with no interferences of innervation zones or tendon, allowing to estimate reliable CV values physiological values. All double differential signals used for CV estimates showed high correlation coefficients (greater than 80%).

**Figure 2:**
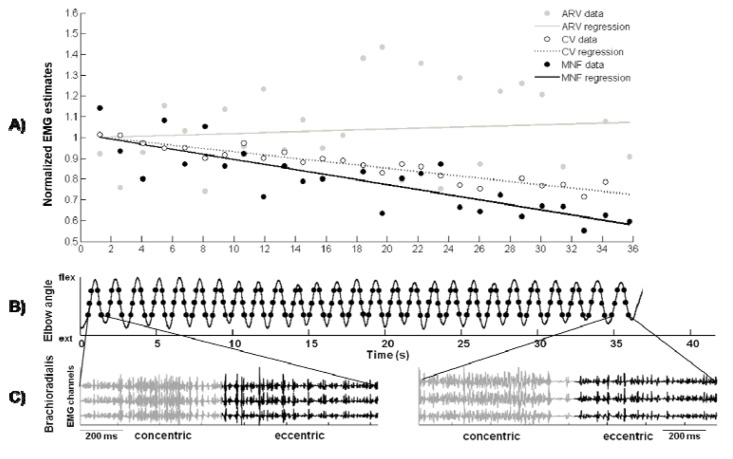
Representative example of recorded signals from brachioradialis during END. B) the time course of the elbow angle is represented. The dots identify the epochs 125 ms long, centered on the time instant corresponding to the crossing of 25%, 50%, and 75% of the elbow ROM, when the EMG variables were estimated. A) Three single differential EMG signals recorded during the first (left) and last (right) pull up are shown. The concentric and eccentric phases are plotted in light grey and black, respectively. Only the channels used for the processing are reported. C) “Fatigue plot” diagram is represented: ARV (grey line), CV (dotted line), and MNF (black line) are normalized with respect to their initial values (that are the intercepts of the regression lines) to compare their percentage changes. The slopes of the linear regressions represent indexes of myoelectric fatigue. In this example, the estimates are calculated at 50% of the ROM in the CON contraction.

Figure 2 shows an example of recorded signals during an END exercise. Figure 2C presents three single differential EMG signals recorded from *brachioradialis* during the first (left) and last (right) pull-up. Figure 2B indicates the time course of the elbow angle with the superimposed dots identifying the epochs in which EMG variables were calculated. Figure 2A shows the so-called “fatigue plot” calculated at the 50% CON phase of that signal: each variable is normalized with respect to its initial value and the slope of the regression line represents an index of myoelectric fatigue.

Normalized rates of changes of angular velocity were found negative (that is significantly different from zero) in END (−0.7 ±0.3%/s). The time-to-task-failure was significantly shorter in END (31 ±10 s) than in ISO (59 ±19 s, p=0.001). Concerning the hip, six participants out of nine increased the hip movement amplitude during the END task.

**Figure 3:**
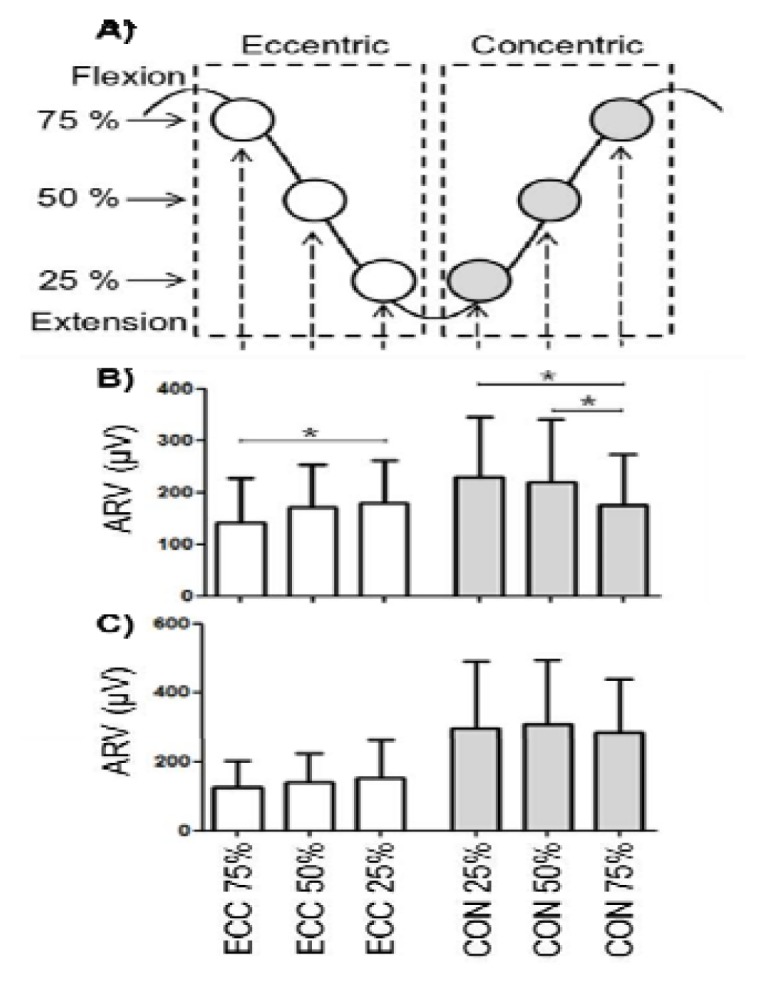
In this diagram, ECC and CON phases are considered separately to detect differences for different angles within each phase and not to compare ECC with respect to CON. The first row represents the six epochs (three for ECC and three for CON) of 125 ms of length, centred on the time instants corresponding to 25–50–75% of the maximum ROM of the elbow joint angle, where 100% corresponded to maximum elbow flexion. Second and third rows show, respectively, the initial values (mean ±SD) of ARV estimated from teres major (top) and brachioradialis (bottom) in the END task. Post hoc significant differences were marked as follows: ^*^ p<0.05

Figure 3 shows ARV estimates in brachioradialis and teres major muscles at the 25%, 50%, and 75% of the elbow ROM in CON and ECC contractions. Teres major showed lower ARV at 75% of elbow flexion in ECC (Friedman test p=0.05) and CON (Friedman test p=0.05), whereas brachioradialis did not present any difference within each phase of the contraction. Moreover, no differences in initial values of CV and MNF were detected throughout the elbow ROM in the two muscles.

Initial values of sEMG variables among contraction phases and tasks are presented in Table 1 (left panel). Concentric contractions in brachioradialis and teres major showed significantly greater ARV than eccentric and isometric contractions. CV did not present any significant differences among contraction phases and tasks.

The normalized slope of sEMG variables and p values of Friedman tests (with post hoc tests, when necessary) are presented in Table 1 (right panel). Overall, END showed a greater rate of fatigue with respect to ISO in both muscles. In particular, the rates of decrease of CV and MNF estimates were found steeper in CON of END than in ISO.

## Discussion

In the herein study an isometric contraction in the hanged position (ISO) and a sequence of dynamic pull-ups (END) until volitional exhaustion were compared in rock climbers. We focused on the EMG activity of two muscles which had never been studied before in rock climbing: brachioradialis and teres major muscles. The main finding was greater fatigue found in the dynamic than in isometric task, both in terms of myoelectric manifestations of fatigue and time to task failure. The second finding was the possibility to properly record physiological CV estimates from these two muscles, in highly dynamic contractions.

The steeper decrease of CV found during END than during ISO (Table 1) highlighted the higher rate of myoelectric manifestations of fatigue in the dynamic task. CV is the physiological variable that better characterizes neuromuscular fatigue, among the measures herein studied. However, in our study ARV and MNF findings were consistent with CV results, indeed we found a time course increase of ARV and a decrease of CV and MNF estimates, as it is usually reported in fatiguing tasks (Merletti et al., 1990). As expected, higher myoelectric fatigue was accompanied with almost 50% shorter time-to-task-failure found in the END (31 ±10 s) than in ISO (59 ±19 s) conditions. The rapid rise in blood lactate levels during exercise in those kind of tasks (Fryer et al., 2011) is responsible for the decrease in muscle pH that is related to the marked CV reduction with fatigue (Brody et al., 1991). As known (Kupa et al., 1995; Mannion et al., 1998) a greater recruitment of type II motor units results in greater myoelectric manifestations of fatigue. For this reason the steeper CV decrease detected in END can be related to a higher recruitment of type II motor units in END with respect to ISO. This finding is related to the higher torque necessary to decelerate and accelerate the body weight with respect to maintain it hanged, leading to an overall greater effort in the dynamic task. However, since we did not measure torque exertions, this explanation remains speculative. Moreover, different contribution of synergistic and antagonist muscles between the isometric and dynamic tasks could affect the indexes of fatigue.

Since this protocol failed to detect difference in CV between contraction types, no assumption can be proposed on motor units recruitment involved. However, ARV estimates detected differences among contraction types allowing to highlight differences in muscle activation. Indeed, ARV provides an overall estimate of muscle activation, since it is related to the number of motor units recruited and their discharge frequency, but the roles played by these two features cannot be distinguished. Accordingly to previous studies (Duchateau and Enoka, 2008; Piitulainen et al., 2013), concentric showed higher muscle activation with respect to the eccentric contractions in both muscles (Table 1). This finding can be explained by: 1) torque exertion greater than body weight during concentric and lower than body weight during eccentric contractions (Duchateau and Enoka, 2008); 2) the greater muscle force that can be expressed during eccentric than concentric contractions (Duchateau and Enoka, 2008), allowing to decrease the activation during eccentric contractions. Obviously, both muscles also showed higher activation during concentric than isometric contractions, since the torque necessary to lift the body (CON) was reasonably higher than that necessary to hold the body hanged.

In the dynamic task muscle activation was found independent of the elbow angle on brachioradialis, whereas teres major showed lower activation at the 75% of the ROM (near to the maximum flexion) with respect to 50% and 25% in both eccentric and concentric contractions. Results on brachioradialis could sound counterintuitive, but we investigated only three epochs in the cycle of pull ups, avoiding the extremes of the ROM (maximum elbow flexion and extension), where the sEMG may be strongly affected by the inversion of the movement. Moreover, the pull up is a complex task, integrating contributions from many synergist and antagonist muscles not herein recorded, thus our findings are not representative to other muscles.

Concerning the hip movement, increased hip oscillations during the END task were observed only in six participants out of nine; thus not an uniform hip strategy was observed among participants to cope with fatigue.

The present study has the following limitations. The sample size was small, making it difficult to generalize and transfer results to the whole population. Our results did not take into account synergic muscle actions, focusing only on two muscles and excluding from the analysis forearm muscles, among others. Moreover, two symmetric tasks were proposed whereas in climbing most arm movements are asymmetrical. Hence, the discussed finding may be biased in understanding real climbing circumstances also for this reason. We did not measure the torque exerted in the two tasks, becoming impossible to associate myoelectric fatigue with the mechanical demand.

In summary, our study extends previous research investigating neuromuscular fatigue on two upper limb muscles, which are pivotal in sport specific settings: brachioradialis and teres major. The main finding was that a sequence of dynamic pull-ups lead to higher fatigue than sustaining the body weight in an isometric condition at half-way of a pull-up (with the elbows flexed at 90°). In particular, the dynamic task was characterized by shorter time-to-task failure and a higher rate of myoelectric manifestations of fatigue than the isometric task.

## Figures and Tables

**Table 1:** Initial values (mean ±SD) and Normalized slope of sEMG variables for brachioradialis (top) and teres major muscles (bottom) during ISO, and for CON and ECC phases of END. EMG variables in END were estimated at 50% of the ROM (both in CON and ECC phases). Friedman test’s p values are shown for each comparison. Post hoc significant differences were marked as follows: ^#^ different with respect to ISO; ^*^ different with respect to END ECC

	Initial values				Normalized slopes (%/s)			

	ISO	END ECC	END CON	p	ISO	END ECC	END CON	p
***Brachioradialis***								
MNF (Hz)	125±20	125±29	133±33	0.18	−0.4±0.3	−0.9±0.7	−1.5±0.4^**^	**0.006**
ARV (μV)	141±86	162±103	318±174^*^^#^	**0.002**	0.4±1.6	1.3±1.6	2.5±1.9^**^	**0.002**
CV (m/s)	4.2±0.3	4.3±0.4	4.4±0.4	0.30	−0.2±0.1	−0.9±0.4	−1.2±0.6^*^	**0.01**
***Teres major***								
MNF (Hz)	90±16	79±21	94±15^#^	**0.01**	−0.5±0.5	−0.7±0.8	−1.6±0.7^*^	**0.04**
ARV (μV)	133±128	169±77	236±125^*^^#^	**0.01**	0.5±0.7	1.5±1.9	1.3±1.3	0.27
CV (m/s)	4.3±0.8	3.4±1.4	3.6±1.2	0.45	−0.4±0.3	−1.5±1.2	−1.8±0.7^*^	**0.04**
